# How intense is high-intensity interval training? Biomarker responses and associations with training load and fitness

**DOI:** 10.1016/j.isci.2025.113738

**Published:** 2025-10-08

**Authors:** Nils Haller, Hannah L. Widauer, Tilmann Strepp, Natalia Nunes, Julia C. Blumkaitis, Mario Wenger, Thomas Stöggl, Lorenz Aglas

**Affiliations:** 1 Department of Sport and Exercise Science, University of Salzburg, Salzburg, Austria; 2 Department of Sports Medicine, Rehabilitation and Disease Prevention, Johannes-Gutenberg-University of Mainz, Mainz, Germany; 3 Division of Exercise and Movement Science, Institute for Sport Science, University of Göttingen, Göttingen, Germany; 4 Department of Biosciences and Medical Biology, University of Salzburg, Salzburg, Austria; 5 Red Bull Athlete Performance Center, Salzburg, Austria; 6 Genetics Division, Department of Morphology and Genetics, Universidade Federal de São Paulo, São Paulo, Brazil; 7 Institute of Pathophysiology and Allergy Research, Center for Pathophysiology, Infectiology and Immunology, Medical University of Vienna, Vienna, Austria; 8 Human Microbiome (HUMI) Research Program, Faculty of Medicine, University of Helsinki, Helsinki, Finland

**Keywords:** Cardiovascular medicine, Kinesiology

## Abstract

High-intensity interval training (HIIT) enhances physical performance but requires close monitoring to avoid illnesses/injuries. We monitored physiological responses at rest during and up to 14 days following a 7-day HIIT intervention to identify chronic physiological changes and to explore correlations between blood biomarkers (blood count, cytokines, creatine kinase [CK], urea, ferritin, and transferrin), training load, cardiorespiratory fitness (VO_2max_), and muscle soreness. Thirty participants were randomly allocated to either HIIT shock cycle (10× HIIT in 7 days) (1) with or (2) without additional low-intensity training after each HIIT session or (3) control group. Repeated HIIT resulted in a chronic decrease of hemoglobin, hematocrit, red blood cells, CK, interleukin [IL]-2, -4, -9, -17A, -17F, tumor necrosis factor alpha (TNF-α), and ferritin. CK showed highest positive correlation with training load and muscle soreness, while VO_2max_ correlated with cytokines IL-5, -6, -10, -17F, -22. The present study revealed reliable biomarkers reflecting training load and VO_2max_, suitable for personalized monitoring of health and recovery and performance optimization.

## Highlights

• Repeated HIIT alters key blood markers linked to health and performance
• Creatine kinase correlates with training load and muscle soreness
• Several cytokines reflect cardiorespiratory fitness
• Biomarkers enable personalized load monitoring of training and recovery

## Graphical abstract

**Figure:**
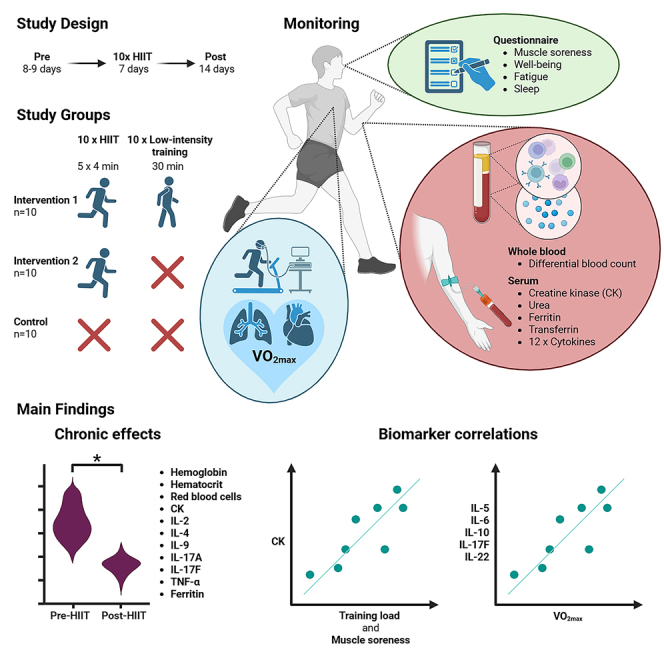

## Introduction

High-intensity interval training (HIIT) is a popular training method to improve endurance performance and overall health.^1^^,^^2^^,^^3^ Intensified training periods, such as HIIT shock microcycles (e.g., 10 HIIT sessions in 7 days^4^), stand out as both popular and demanding training regimes, known for their effectiveness in improving athletic performance.^5^^,^^6^^,^^7^ However, these strenuous exercise regimens with minimal recovery phases require careful monitoring due to their potential to increase the risk of injury, overtraining, or illness.^8^ Non-functional overreaching or overtraining (i.e., performance decline despite maintaining or intensifying training load^8^^,^^9^) may result from training errors and insufficient recovery phases.^10^ On a tissue level, injuries (e.g., tendinopathy and bone stress injuries) may result from excessive microdamage when the magnitude of loading (i.e., intensity, frequency, and duration) exceeds the tissue’s current load-bearing capacity or when recovery between sessions is inadequate.^8^ In addition, the incidence of illnesses such as common infections (e.g., upper respiratory tract infections) was associated with excessive training load^11^^,^^12^ as well as participating in many competitive events.^13^ These associations have predominantly been observed in well-trained or athletic populations.

HIIT shock microcycles may therefore serve as a model to study the effects of intensified competitive training periods with minimal recovery periods, potentially shifting athletes along a continuum from homeostasis toward overreaching.^8^^,^^9^^,^^10^ Blood-based biomarkers (as indicators of normal biological or pathogenic processes, or responses to an exposure^14^) may help to detect this shift.^15^ They offer critical tissue-specific insights into the physiological response to intense training periods, thereby enabling early indications of fatigue and overreaching. Biomarkers also provide diagnostic or prognostic value for fatigue, injury, illness, or nutritional deficiencies, thus offering individual insights into health and performance.^15^

In this respect, certain biomarkers, such as cytokines, have been associated with overtraining.^16^^,^^17^ Cytokines are important mediators regulating various inflammatory as well as anti-inflammatory immune processes^18^ and are affected by regular exercise. Therefore, there has been growing interest among researchers in monitoring cytokine levels,^19^ particularly interleukin (IL)-1, IL-6, IL-10, and tumor necrosis factor alpha (TNF-α), which are commonly elevated after acute exercise.^19^^,^^20^^,^^21^^,^^22^ During exercise, IL-6, which is usually attributed to pro-inflammatory actions, elicits anti-inflammatory effects when it is secreted by contracting skeletal muscles.^23^ In contrast, typical pro-inflammatory cytokines that increase during viral infections such as IL-1β remain rather constant during exercise.^24^^,^^25^ TNF-α and IL-4 are key players in energy metabolism and glucose homeostasis,^26^^,^^27^^,^^28^ and both are modulated by exercise, with decreases reported for TNF-α and increases for IL-4.^29^^,^^30^ Other cytokines such as IL-2, IL-9, and IL-17 have rarely been studied in the context of exercise. IL-2 is secreted from activated and proliferating immune cells, mainly T lymphocytes,^31^ IL-9 mediates the protection against parasitic infections,^32^ and IL-17 is involved in cuing inflammatory reactions in response to infection, injury, and physiological impacts as well as offering protection at barrier surfaces.^33^

While individual cytokines, such as those involved in the inflammatory response, have shown potential in monitoring specific physiological domains during exercise, a holistic set of biomarkers might provide a more comprehensive assessment of the exercise response. This is emphasized by the multisystemic nature of responses to exercise, encompassing indicators of muscle damage, metabolism, and immune response. In the present study, a longitudinal, systematic evaluation of 32 biomarkers over a 1-month period was conducted, including 12 cytokines (IL-2, IL-4, IL-5, IL-6, IL-9, IL-10, IL-13, IL-17A, IL-17F, IL-22, interferon [IFN]-γ, and TNF-α), markers of muscle damage (creatine kinase, CK) and protein metabolism (urea), iron homeostasis parameters (ferritin and transferrin), as well as 16 different differential blood count parameters (lymphocytes, LYMs; granulocytes, GRs; GR%; white blood cells, WBCs; monocytes, MO%; LYM%; platelets, PLTs; red blood cells, RBCs; hemoglobin, HGB; hematocrit, HCT; red blood cell distribution width, RDWCV; platelet distribution width, PDW%; mean platelet volume, MPV; mean corpuscular hemoglobin; mean corpuscular hemoglobin concentration; and mean corpuscular volume, MCV). The objectives of the study were (1) to monitor longitudinal changes in blood-based biomarkers at rest during and up to 14 days after an exhausting 7-day HIIT shock microcycle intervention, (2) to evaluate chronic changes in biomarkers (pre compared to 7–14 days post intervention), and (3) to assess correlations between biomarkers and measures for training load, cardiorespiratory fitness (VO_2max_), and self-reports on muscle soreness and fatigue. This comprehensive approach enables us to gain valuable insights into physiological responses to a highly demanding training period, ultimately contributing to the identification of biomarkers suitable for monitoring athlete health and performance—a crucial step toward designing personalized, effective training programs to maximize health and performance.

### Study overview

**Figure 1:**
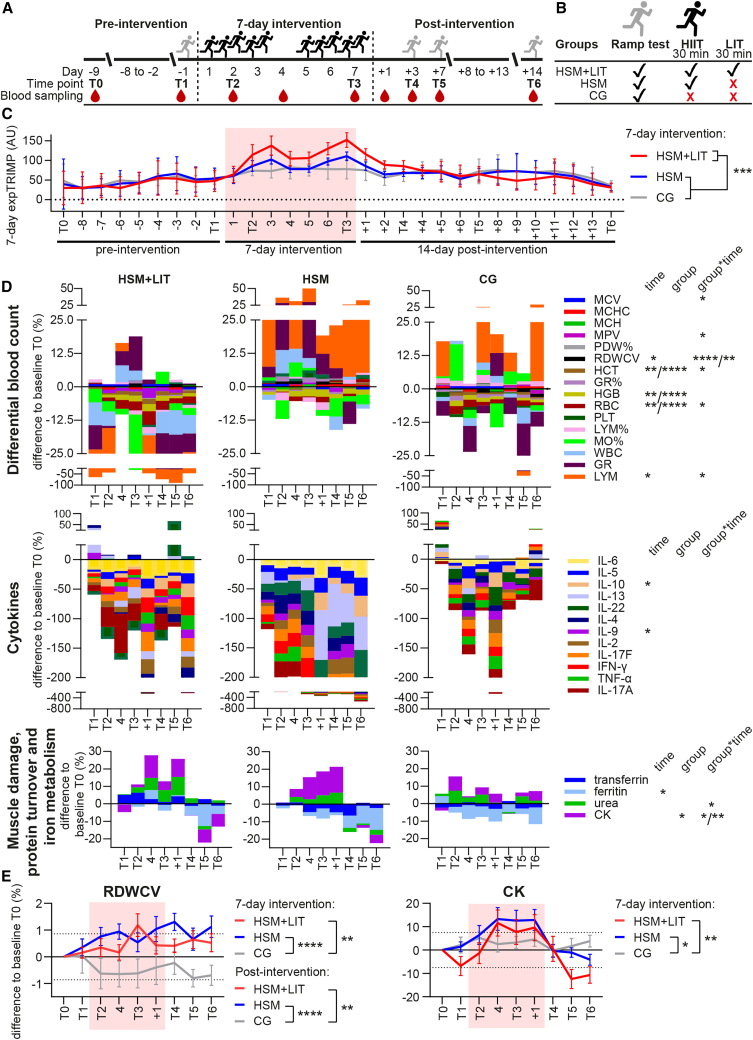
Study design, HIIT training load, and longitudinal biomarker changes (A and B) Study design and (B) participant allocation to training protocols. The HSM+LIT and the HSM group performed ten HIIT sessions over 7 consecutive days, indicated by a black runner, with an additional 30-min LIT in the HSM+LIT group. CG kept their regular training regimens. On days 2, 3, 6, and 7 of the intervention, two HIIT sessions were performed, one in the morning and one in the afternoon. At T1, T4, T5, and T6, physiological exercise testing was conducted (ramp test, gray runner). Blood was collected at rest prior to each exercise session on T0, T1, T2, T3, T4, T5, and T6, at day 4 of the intervention and 1 day post intervention (+1). Numbers with a minus represent days before the 7-day intervention period, numbers with a plus represent the days after, and numbers without plus or minus refer to days within the 7-day intervention. (C) TRIMP was weighted exponentially over the last 7 days to account for the influence of previous exercise. Data are shown as mean with SD. (D) Investigated biomarkers shown as the median of the percentage difference from the baseline T0 for each group and every biomarker. Statistically significant effects were determined with restricted maximum likelihood mixed-effects analysis and indicated as ∗*p* < 0.05, ∗∗*p* < 0.01, ∗∗∗*p* < 0.001, ∗∗∗∗*p* < 0.0001. (E) Percentage changes from baseline T0 for representative biomarkers with significant group∗time interactions shown as mean with SEM. Dotted lines represent the SD threshold calculated for biomarker values at T0 and T1 of all 30 participants. HSM, high-intensity interval training shock microcycle; HSM+LIT, HSM and additional 30-min low-intensity training; CG, control group.

The study spanned 4 weeks for all participants, starting with an 8- to 9-day pre-intervention phase, followed by a 7-day intervention period, and concluding with a 14-day post-intervention phase. A total of 30 participants were included and randomly allocated to one of three groups: either group (1) HIIT shock microcycle consisting of 10 HIIT sessions (HSM), (2) HSM + additional 30-min low-intensity training (HSM+LIT) after each HIIT session (resulting in 300 min [i.e., +75%] of additional exercise time), or (3) a control group (CG; *n* = 10 per group), proceeding with their usual training regimen (Figures 1A and 1B; Table 1).

**Table 1:** Anthropometric data and physiological characteristics of the study participants at time point T1 (entry physiological exercise testing)

	HSM+LIT	HSM	CG
Athletes (n)	10	10	10
Male (n)	9 (90%)	6 (60%)	8 (80%)
Female (n)	1 (10%)	4 (40%)	2 (20%)
Age (years)	28.6 ± 6.4	29 ± 5.4	29.2 ± 6.4
Height (cm)	179 ± 7	173 ± 8	178 ± 7
Weight (kg)	71.3 ± 7.9	65.0 ± 8.0	71.0 ± 9.8
BMI (kg/m^2^)	22.2 ± 2.0	21.7 ± 2.4	22.1 ± 2.1
VO_2max_ (ml·min^−1^·kg^−1^)	60.3 ± 5.2	60.3 ± 6.9	59.5 ± 2.6

Data are shown as mean with SD. HSM, high-intensity interval training shock microcycle; HSM+LIT, HSM and additional 30-min low-intensity training; CG, control group.

The rationale for selecting these specific protocols lies in their alignment with existing literature and practical considerations in endurance training. Long aerobic intervals (i.e., 5 × 4 min HIIT) were chosen as these intervals effectively improve VO_2max_.^6^^,^^34^ The inclusion of the HSM+LIT group allows for the evaluation of potential dose-response relationships by investigating the effects of increased low-intensity exercise time on biomarkers and physiological adaptations compared to a standard HIIT shock cycle. In addition, anecdotal concerns from practitioners suggest that reduced low-intensity training (LIT) during an HIIT shock cycle might negatively affect performance, as LIT plays a crucial role in stimulating physiological adaptations in endurance athletes. Moreover, as outlined in the introduction, the short overreaching period of the HIIT shock cycle provides an opportunity to investigate both acute and chronic physiological responses, including recovery kinetics over a 2-week period.

## Results

### Training load and longitudinal changes in biomarkers

The 7-day exponentially weighted training impulse (expTRIMP), as a readout for training load based on heart rate and exercise duration, increased from pre-intervention to the intervention period (*p* < 0.001) in all groups. In addition, a significant difference between HSM+LIT and CG during the intervention phase was found (*p* < 0.001, Figures 1C and S1). During the intervention, the expTRIMP was significantly higher for HSM+LIT (116 ± 33 AU) compared to HSM (87 ± 20) and CG (75 ± 21).

Regarding blood biomarkers, significant group∗time interaction effects in comparison to CG were observed for differential blood count parameters RBCs, HCT, MCV, LYMs, RDWCV, and MPV, as well as for CK and urea (Figures 1D and S2; Table S1). In particular, during the intervention period, RBCs, HCT, MCV, RDWCV, and CK and MPV, RDWCV, CK, and urea were significantly altered compared to the CG in HSM and HSM+LIT, respectively. Post intervention, RDWCV was different in both intervention groups compared to CG, and MPV and LYMs only in the HSM+LIT group. For HSM and HSM+LIT, RDWCV increased on average by 0.7% (difference to baseline T0) during the intervention and remained high even during post intervention, while CG was around or fell below baseline levels (Figure 1E). Considering the average of all subjects, CK increased during the intervention (9%–11% difference to baseline) and decreased 7–14 days post intervention to 11.5% and 2.6% below baseline (T0) in HSM+LIT and HSM, respectively, with no notable changes in CG. In addition, for HGB as well as cytokines IL-9 and IL-10, and ferritin, significant changes over time compared to pre-intervention were found (*p* < 0.05, Table S2).

### HIIT-induced chronic effects on blood biomarkers

**Figure 2:**
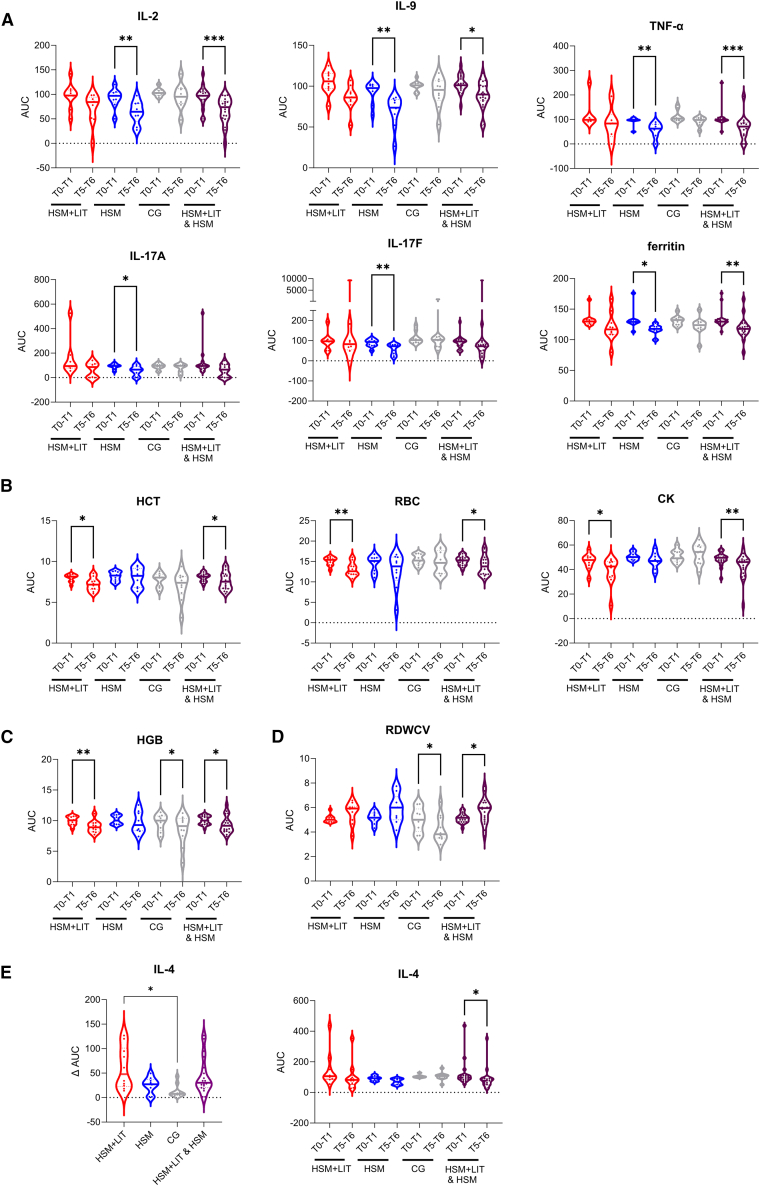
Chronic effects of HIIT shock microcycle on biomarkers Chronic effects were observed by comparing the AUC of the difference to baseline values from pre- (T0-T1) and post intervention (T5-T6) of each participant and occurred in (A) HSM, (B) HSM+LIT, (C) HSM+LIT and CG, (D) CG, or (A–E) when pooling HSM and HSM+LIT. (E) IL-4 was the only biomarker where a significant difference was detected for the ΔAUC, defined as difference between AUC (T0-T1) and AUC (T5-T6). ∗*p* < 0.05, ∗∗*p* < 0.01, ∗∗∗*p* < 0.001.

Comparing the area under the curve (AUC) pre- (T0-T1) and 7–14 days post intervention (T5-T6), 12 of the 32 (37.5%) investigated blood biomarkers exhibited chronic changes in HSM, HSM+LIT, and the pooled group HSM and HSM+LIT (Figures 2A–2E and S3). In HSM+LIT, HCT, RBCs, CK, and HGB decreased significantly by 11%–18.2% (*p* = 0.004–0.012), whereas in HSM, ferritin decreased by 9%, and IL-2, IL-9, TNF-α, IL-17A, and IL-17F by 22.2%–36.8% (*p* = 0.002–0.014, Table S3). In the HSM+LIT and HSM pooled group, all aforementioned biomarkers also decreased, except IL-17A and IL-17F. However, HGB decreased similarly in CG (8.9%) as in HSM+LIT (11.5%) and the pooled HIIT group (8.5%), indicating a likely HIIT-independent effect. Pooling both HIIT groups revealed a significant 15.4% increase in RDWCV (*p* = 0.031), contrasting with a 23.6% decrease in CG (*p* = 0.042). Additionally, only in the pooled group, a 20.7% reduction in IL-4 was observed. When analyzing ΔAUC (difference in AUC between T0-T1 and T5-T6), IL-4 was significantly higher in HSM+LIT compared to CG (*p* = 0.014).

### Biomarkers for training load, muscle soreness, and cardiorespiratory fitness

**Figure 3:**
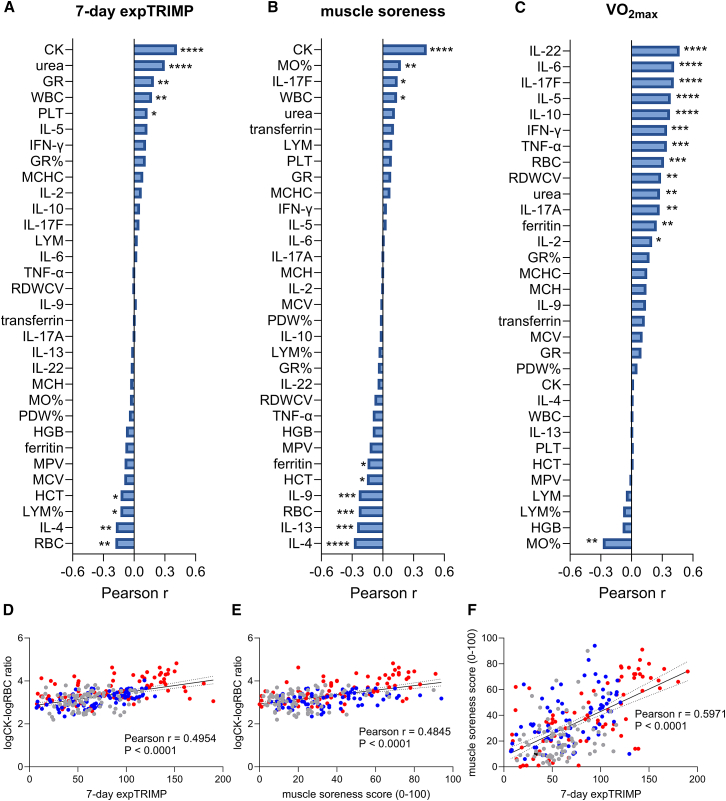
Ranking of biomarker correlations Ranking of Pearson r of 32 biomarkers correlating with (A) 7-day expTRIMP, (B) muscle soreness score, and (C) VO_2max_, according to the Pearson correlation matrix and combining data of all 30 participants. Correlations between the logCK-to-logRBC ratio with (D) the 7-day expTRIMP (TRIMP weighted exponentially over the last 7 days to account for the influence of previous exercise) or (E) the muscle soreness score, and with the 7-day expTRIMP with the muscle soreness score (F). HSM+LIT, red; HSM, blue; CG, gray. ∗*p* < 0.05, ∗∗*p* < 0.01, ∗∗∗*p* < 0.001, ∗∗∗∗*p* < 0.0001.

A Pearson correlation matrix was used to assess associations between biomarkers, self-reported assessment data, and training load (expTRIMP). Significant positive moderate (CK, r = 0.42; urea, r = 0.3) to weak correlations (GR, WBCs, and PLTs, r = 0.13–0.19) with the expTRIMP were observed. Conversely, RBCs, IL-4, LYM%, and HCT displayed weak negative correlations with the training load (r = −0.18 to −0.13, Figures 3A, S4, and S5A). Muscle soreness was most strongly positively associated with CK, MO%, IL-17F, and WBCs (r = 0.43–0.14), while IL-4, IL-13, RBCs, IL-9, HCT, and ferritin showed negative correlations (r = −0.28 to −0.15, Figures 3B, S4, and S5B). Regarding VO_2max_, positive correlations were observed for IL-22, IL-6, IL-17F, IL-5, IL-10, IFN-γ, TNF-α, RBCs, RDWCV, urea, IL-17A, ferritin, and IL-2 (r = 0.47–0.2), while MO% was negatively correlated (r = −0.28, Figures 3C and S6). The strong correlation among the investigated cytokines with VO_2max_ is further highlighted by clustering together in the network analysis (Figure S7).

The ratio of the biomarker showing the highest positive (CK) and the highest negative correlation (RBCs) with the expTRIMP was calculated. Pearson r of this logCK-to-logRBC ratio increased (r = 0.5) compared to the individual parameters (r = 0.44 for CK and −0.15 for RBCs). Since expTRIMP highly correlated with muscle soreness (r = 0.60), the logCK-to-logRBC ratio also correlated with the muscle soreness score as depicted by the augmented Pearson r value (from r = 0.42 for CK and r = −0.19 for RBCs to r = 0.48 for the ratio, Figures 3D–3F).

**Figure 4:**
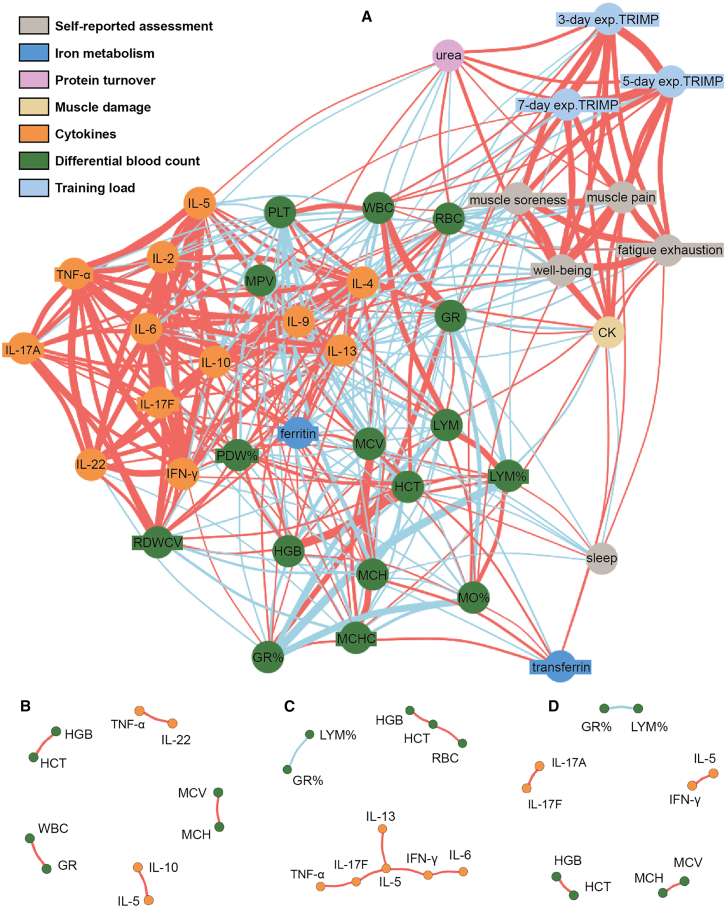
Interconnectedness of biomarkers A Network including data of all 30 participants and all 32 biomarkers, the expTRIMP, as well as the self-reported assessment dataset. Thickness of lines represents the magnitude of correlation of two parameters. Red lines representing positive correlations, blue lines negative correlation. Knots are only connected if significant correlations were observed. Top ten correlating biomarkers for each group, HSM+LIT (B), HSM (C), and CG (D).

When correlating all biomarkers and self-reports, two major interconnected clusters became evident: cluster 1 comprising the parameters for training load, as well as the scores for muscle soreness, muscle pain, fatigue, and well-being, and their correlation with CK and urea and cluster 2 mainly including cytokines, ferritin, and the differential blood count parameters PLTs, MPV, RDWCV, and PDW% (Figure 4A). Regarding cluster 2, IL-22 correlated positively with IL-4, IL-9, IL-13, and IL-17A (r = 0.44–0.52). In addition to IL-22, IL-4 showed a positive correlation with IL-9 and IL-10 (r = 0.47 and 0.45, respectively). IL-17A correlated positively with IL-22, IL-2, IL-6, and IFN-γ (r = 0.40–0.52). IL-17F displayed positive correlations with IL-6, IL-5, IL-2, and TNF-α (r = 0.40–0.45). IFN-γ correlated positively with RDWCV and IL-9 (r = 0.42 and 0.43, respectively). In addition to IL-17F, TNF-α showed a positive correlation with RDWCV (r = 0.46). IL-9 displayed positive correlations with IL-5, IL-4, IL-22, IFN-γ, and IL-2 (r = 0.4–0.48). A positive correlation was also observed for RDWCV with IL-2 (r = 0.44).

When separating the top ten correlating biomarkers for each group (Figures 4B–4D), a strong positive association between GR and WBCs (r = 0.92 in HSM+LIT) or a strong negative correlation with LYM% (r = −0.88 in HSM and r = −0.96 in CG) was observed in all groups. In HSM+LIT, TNF-α highly correlated with IL-22 (r = 0.95) and IL-5 with IL-10 (r = 0.91). In the HSM group, a cluster of strongly correlating pro-inflammatory cytokines, namely TNF-α, IL-17F, IL-5, IFN-γ, IL-13, and IL-6 (r = 0.89–0.95), was found. Interestingly, these cytokine clusters were not observed in the CG.

## Discussion

To our knowledge, this is the first study to examine chronic physiological responses using a comprehensive biomarker panel within a standardized 7-day HIIT shock microcycle over 4 weeks. Differential responses across the study period were observed for WBCs, HCT, MCV, GR, RDWCV, MPV, TNF-α, urea, and CK indicating their sensitivity to the strenuous HIIT intervention. Both HIIT interventions resulted in chronic decreases in IL-2, IL-4, IL-9, TNF-α, ferritin, HCT, CK, and RBCs, while RDWCV increased. CK showed the strongest correlation with training load and muscle soreness according to the Pearson correlation matrix combining data of all three groups, while RBCs displayed the strongest negative correlation with training load. Based on these findings, we identified the logCK-to-logRBC ratio as a combined biomarker, offering a more robust correlation with training load and muscle soreness than the individual parameters.

Regarding chronic effects occurring due to repeated HIIT, nine blood biomarkers chronically changed in pooled intervention groups, namely IL-2, IL-4, IL-9, TNF-α, ferritin, HCT, CK, RBCs, and RDWCV. Since HGB also decreased chronically in the CG, we exclude this parameter as an HIIT-sensitive biomarker. A reduction in HGB, HCT, and RBCs has also been observed in professional cyclists during periods of intensified training^35^^,^^36^ and can be attributed to the increase of plasma volume frequently described following intensive training periods.^37^ The increase in plasma volume is a compensatory mechanism to prevent exercise-induced hemoconcentration.^35^ Another reason might be hemolysis of primarily senescent RBCs due to mechanical forces induced by heavy running or as RBCs pass capillaries of contracting muscles. In contrast to common assumptions that a decrease in RBCs might be disadvantageous for athletes, hemolysis especially of aged or senescent cells increases the amount of juvenile RBCs, which possess an improved oxygen transfer capacity and in turn improve oxygen supply to tissues and the aerobic capacity of athletes.^38^

RDWCV significantly increased chronically in the pooled HIIT groups compared to CG, where RDWCV even decreased. RDWCV is a parameter describing the variability of RBC size, and increased levels thereof indicate higher cell size variability, potentially attributed to impaired erythropoiesis or a decreased life span of RBCs.^39^ Increases in RDWCV were also reported in runners 4 and 24 h after completing a marathon^40^ as well as after an intensified training phase in elite kayakers.^41^ However, since the herein reported RDWCV values were within the reference range (11.5%–15.5%^42^), it is unlikely that HIIT induced negative effects on RBC life span and erythropoiesis.

Ferritin is storing high amounts of iron that would otherwise be toxic to cells.^43^ In HSM, ferritin was chronically reduced. Low and even suboptimal ferritin levels have been reported for elite athletes and recreationally active individuals across a variety of sports compared to sedentary controls.^44^^,^^45^^,^^46^ This decrease can be explained either by increased iron utilization, elevated production of iron-dependent proteins such as HGB, or loss through augmented sweating.^46^ A reduction in HCT,^47^ RBCs, HGB,^48^^,^^49^^,^^50^ and potentially ferritin levels accompanied by an increase in RDWCV might therefore be a typical response 7–14 days following intensive endurance exercise and, thus, reflects ongoing recovery processes rather than a chronic maladaptation. However, if these alterations persist, they could indicate iron deficiency and anemia, warranting dietary supplementation. Regular monitoring of these biomarkers following intense workout regimens is therefore recommended.

During the intervention, IL-2, IL-9, IL-17A, IL-17F, TNF-α, and ferritin also chronically decreased. Since TNF-α negatively affects glucose metabolism^51^ and induces several molecular pathways, which lead to skeletal muscle loss,^52^ the chronic reduction in the HSM group might be associated with accelerated energy homeostasis to maintain muscle mass. Baj et al.^53^ observed a decreased IL-2 production by lymphocytes in male cyclists undergoing a 6-month intensive training and racing season. IL-2 promotes clonal expansion, differentiation, and memory formation of T effector cells and, thus, plays an important role in immune regulation and homeostasis.^54^ In our case, the reduction in IL-2 concentrations might counteract exercise-induced inflammatory responses by dampening effector T cell functions. IL-4 is implicated as a positive regulator of glucose homeostasis by promoting insulin-induced glucose uptake and increasing insulin sensitivity.^55^^,^^56^ In our study, the reduction in IL-4 likely results from the enhanced energy demands due to the HIIT shock cycle. IL-4, IL-9, and IL-17 are key cytokines involved in allergic and autoimmune responses.^57^^,^^58^^,^^59^ In this respect, lowering IL-4 would be beneficial in reducing such inflammatory responses.^60^ IL-4 plays a role in wound healing and tissue repair by activating fibroblasts and supporting collagen synthesis.^61^ Reduced levels in serum likely arise from the increased consumption required for these mechanisms in the tissue after the exercise. Overall, the decrease in cytokine levels 7–14 days post exercise likely reflects the resolution of exercise-induced inflammation and the completion of the recovery process. However, in case the reduction in cytokine levels persists, it may point to exercise-induced immunosuppression posing negative effects on the athlete such as an increased susceptibility to infections.^62^ Regular, long-term monitoring of these cytokines is therefore recommended and could provide valuable insights into recovery dynamics and training adaptations.

CK is commonly used as a surrogate variable for muscle damage and physical strain since it is released from skeletal muscle fibers during intense exercise, such as HIIT.^63^ Usually, CK is increased in response to prolonged strenuous exercise and remains elevated for several days. However, the magnitude of CK increases varies notably in studies.^64^^,^^65^ Repeated bouts of exercise or regular training have been shown to reduce the risk of sudden muscle injury, accompanied by a decrease in CK levels, likely due to the strengthening of cellular structures and muscle fibers.^65^^,^^66^ In line with these findings, Behringer et al.^67^ found that after a repeated bout of eccentric exercise in healthy males, CK enzyme activity decreased alongside lower CK concentrations after the second bout of exercise. This indicates that the chronically decreased CK levels, which we have observed post intervention, might be explained by muscle adaptation or a repeated bout effect. While observed chronic changes may suggest improvements regarding metabolic health, it remains uncertain if these changes will have lasting effects or are just temporary and will revert to baseline over time.

Regarding the determination of serum biomarkers representative for training load, muscle soreness, and cardiorespiratory fitness, we found that CK and urea show the highest correlations with training load, CK and MO% with muscle soreness, and the cytokines IL-22, IL-6, IL-17F, IL-5, and IL-10 with VO_2max_. Of note, while training load showed a strong correlation with muscle soreness, CK correlated with both but did not serve as a specific indicator for either condition. In contrast, urea and MO% were more specific markers for training load and muscle soreness, respectively. Urea is a major product of protein breakdown that increases in response to exercise, which can be explained by increased energy utilization and the concomitant depletion of glucose storages, resulting in protein degradation for gluconeogenesis. As a result, it is widely used as a marker of fatigue in athletes.^63^ Still, we observed that CK displayed the strongest positive correlation with expTRIMP, while RBCs had the strongest negative correlation. Upon combining these two biomarkers in the logCK-to-logRBC ratio, the correlation coefficient increased, which can partly be explained by occurrence of hemolysis due to the intensive training,^38^ which reduces RBC numbers. Incorporating the RBC count into the ratio enhances the robustness of CK levels, reducing the influence of hemolysis. Using combined biomarkers, such as the testosterone-to-cortisol ratio or the CK-to-LDH ratio, is a common practice in sports science and exercise physiology.^68^

The observed correlations of VO_2max_ and cytokines were unexpected, as previous studies have reported negative correlations for cytokines like IL-6 and IL-8 with VO_2max_.^69^^,^^70^^,^^71^^,^^72^ Furthermore, markers of systemic inflammation, such as pro-inflammatory cytokines, have been shown to be inversely correlated with both VO_2max_ and the overall fitness status.^73^ In contrast, a study with ten male participants examining the effects of exercise intensity during a 60-min treadmill running workout found a positive association between IL-6 and VO_2max_.^74^ In a cross-sectional study including 52 people, a similar positive correlation (r = 0.31) between VO_2max_, as determined by the Rockport aerobic test, with IL-10 at rest was observed as in our study.^75^ We observed a positive correlation with VO_2max_ not only for IL-6 and IL-10 but also for IL-22, IL-17F, and IL-5. Especially during prolonged exercise, IL-6 and IL-10 play key regulatory roles in maintaining blood glucose levels by gluconeogenesis and influencing the energy metabolism.^76^ In the liver, IL-6 increases the expression of the gluconeogenic enzymes phosphoenolpyruvate carboxykinase and glucose-6-phosphatase and promotes lipolysis in adipose tissue. In contrast, IL-10 rather has a supportive role and is required to balance the inflammatory effects of IL-6 and other cytokines to mitigate excessive inflammation and potential tissue damage. In respect of energy metabolism, it is sufficient to state that the correlations between IL-6 and IL-10 with VO_2max_ are reasonable. The associations between VO_2max_ and cytokines suggest a potential role for cytokines in modulating energy production and consumption. This effect is complemented by an enhanced anti-inflammatory response, which helps to limit excessive inflammation, prevent diseases, and protect against tissue damage. Through these mechanisms, cytokine-driven immunomodulation contributes to overall metabolic health, particularly evident during high-intensity training regimens.

Taken together, these results contribute to the understanding of exercise-induced physiological responses by identifying specific cytokines and other biomarkers that change in response to HIIT and correlate with subjective assessments. This represents a key first step in biomarker identification for practical applications.^77^ The next step will be to explore these biomarkers in longitudinal studies to test their reliability and applicability in longer training settings. By correlating biomarkers with self-reported measures, we can gain deeper insights into the relationship between physiological responses and subjective experiences of fatigue, soreness, and recovery. The ultimate goal is to develop a panel of tissue-specific biomarkers (e.g., for immune response, muscle soreness, and inflammation), ideally complemented by sensitive questionnaire items, to enable personalized training and recovery prescriptions. Such approaches have the potential to enhance individual performance, reduce injury risk, and support precision training in both athletic and clinical populations.

### Practical application and concluding remarks

While pro-inflammatory cytokines such as IL-6 and TNF-α usually increase post exercise and then return to baseline within 24–72 h post exercise,^19^^,^^20^^,^^21^^,^^22^ we identified 11 biomarkers (IL-2, IL-4, IL-9, IL-17A, IL-17F, TNF-α, ferritin, HCT, CK, RBCs, and RDWCV) that changed chronically (7–14 days post exercise) over the course of the study. In addition, certain biomarkers were found that correlated specifically with training load (CK, urea, RBCs, and the logCK-to-logRBC ratio) and/or muscle soreness (CK and MO%) or cardiorespiratory fitness (IL-22, IL-6, IL-17F, IL-5, and IL-10). We are the first to show that certain cytokines are correlating with an athlete’s cardiorespiratory fitness, which are, thus, promising novel biomarker candidates for monitoring energy metabolism, (anti-) inflammatory responses, and the overall metabolic health in athletes. The downside of routine monitoring of cytokines is their high inter- and intra-subject variability, low-cost efficiency, and an elaborate measurement routine thus far. However, with advances in technology, cytokines may become more relevant to practitioners through the use of point-of-care devices.^77^

In summary, we recommend using a set of biomarkers that represent specific exercise metrics to ensure effective load management and recovery. This approach aims to provide a holistic view of an athlete’s metabolic health and performance, potentially enabling the optimization and individualization of training regimes to achieve peak performance.

### Limitations of the study

Limitations of the present study include the unequal biological sex distribution within and between the groups (10%–40% females), making biological sex-specific interpretations difficult as well as the relatively small number of participants (*n* = 10 per group). To compensate for the limitation of a small sample size, we employed a longitudinal design with repeated measurements at multiple time points. This strategy was intended to maximize the informational value of the data and to provide a more comprehensive analysis of temporal trends. Our findings are specific to trained individuals, potentially reducing applicability to the general population. On the other hand, such intensified training interventions aim at improving endurance performance especially in already trained individuals.^4^

The randomization after T1 in our study may have unintentionally increased participants’ training loads in the CG, potentially due to the so-called Hawthorne effect,^78^ where being part of a study motivates individuals to alter their usual training behavior, such as increasing physical activity levels. This effect may have been intensified by repeated performance assessments, serving as “goal setting” after the intervention period, which could have prompted behavior changes and led to increased training volumes in the CG.^79^ Finally, the present study focused on blood sampling at rest, where only small changes in biomarker concentrations are expected to occur compared to control treatments.^80^ The identification of significant biomarker effects and correlations even under these resting conditions strengthens the validity of our findings.

An additional limitation of our study is that we did not control or assess the hydration and nutrition status of the subjects during the intervention, which can be considered a realistic training scenario but might also impact the interpretation of biomarker measurements. Subjects were, however, advised to arrive fully nurtured (keeping their regular dieting regimes) and in a properly hydrated condition.

## Resource availability

### Lead contact

Requests for further information and resources should be directed to and will be fulfilled by the lead contact, Nils Haller (nhaller@uni-mainz.de).

### Materials availability

This study did not generate new unique reagents.

### Data and code availability

This study did not generate or analyze standardized datasets (e.g., sequencing, proteomics, or imaging data). The data supporting the findings of this study consist of physiological and performance measures and are available from the lead contact upon reasonable request. No custom code was used.

## Acknowledgments

The authors sincerely thank all participants for their valuable contributions. The authors thank Prof. Fatima Ferreira for providing lab space and equipment. The authors gratefully acknowledge Dr. Ferdinand Aglas for providing the Eurolyser system.

The graphical abstract was created in BioRender. Aglas, L. (2025) https://BioRender.com/0osfbtk.

The study is a cooperation project between the University of Salzburg, the University of Mainz, and the Red Bull Athlete Performance Center. The study received funding from the Red Bull Athlete Performance Center for the scientific accompaniment of the monitoring concept in the context of which data for the current study were collected. H.L.W., L.A., M.W., and N.N. received support from the Austrian Science Funds (FWF) Projects I5312 and P34207 https://doi.org/10.55776/I5312 and https://doi.org/10.55776/P34207.

## Author contributions

N.H.: initiation, study design, supervision, concept, formal analysis, writing – original draft, and writing – review and editing. H.L.W.: investigation, data analysis and figure preparation, formal analysis, visualization, and writing – original draft. T. Strepp: TRIMP data, formal analysis, investigation, and data curation. N.N.: formal analysis and visualization. J.C.B.: investigation and data curation. M.W.: investigation and formal analysis. T. Stöggl: initiation, study design, funding acquisition, review, and writing – review and editing. L.A.: initiation, supervision, data analysis and figure preparation, review and editing, concept, funding acquisition, visualization, writing – original draft, and writing – review and editing. All authors read and approved the manuscript.

## Declaration of interests

T. Strepp and T. Stöggl are employees of the Red Bull Athlete Performance Center.

## STAR★Methods

### Key resources table

**Table:** 

REAGENT or RESOURCE	SOURCE	IDENTIFIER
**Antibodies**		

Mouse monoclonal anti-human ferritin	Bio-Rad	Clone F23 [7A4], Cat# 4420-3010; RRID: AB_617278
Mouse monoclonal anti-human transferrin	R&D Systems	Clone 507506; Cat# MAB5746; RRID: AB_2287232
rabbit polyclonal anti-human ferritin	Sino Biological	Cat# 12482-T16; RRID: AB_3676889
rabbit polyclonal anti-human transferrin	Biozol	Cat# SIN-11019-RP02; RRID:AB_3095074
horseradish peroxidase-conjugated recombinant alpaca monoclonal anti-rabbit IgG	Creative Diagnostics	Clone IB21, Cat# CABT-L4215

**Chemicals, peptides, and recombinant proteins**		

Recombinant human transferrin	Abcam	Cat# ab83560
Native human ferritin	Bio-Rad	Cat# 4420-4804

**Critical commercial assays**		

Human Th Cytokine Panel (12-plex) with V-bottom or filter plate V02	BioLegend	Cat# 741027 and 741028, Lot# B315001/B338131/B327881

**Software and algorithms**		

GraphPad Prism Version 9.1.2	–	–
R Studio Version 4.4.1	–	–
SPSS 27	–	–

**Other**		

BD Vacutainer® Serum Tubes	BD	SKU: 367820 GTIN: 00382903678204
BD Vacutainer® Heparin Tubes	BD	SKU: 367874 GTIN: 00382903678747
Global Navigation Satellite System (GNSS) watch	Garmin	Forerunner 935
Heart rate (HR) chest strap	Garmin	HRM Pro
Treadmill	HP Cosmos	Saturn
Breath-by-breath gas analyzer system	Cosmed	Quark CPET
Lactate measurement	EKF diagnostic GmbH	Biosen S-line Clinic
Differential blood Celltac	Nihon Kohden	MEK 6400 system
Flow cytometry	Beckman Coulter	Cytoflex S
Eurolyser	Eurolyser Diagnostica GmbH	CCA180

### Experimental model and study participant details

#### Ethics statement

This study was registered on ClinicalTrials.gov (identifier: NCT05067426). Ethical approval was granted by the local ethics committee of the University of Salzburg (GZ 02/2021), with all procedures complying with the standards of the Declaration of Helsinki.

### Experimental design

A total of 30 participants were included and randomly allocated to one of three groups with *n* = 10 per group (Table 1). Participants were either runners or athletes with a considerable amount of running (>50 km·wk−1) in their regular training. Eligible individuals were between 18 and 45 years of age and had to meet one of the following criteria: VO_2max_ of ≥50 mL min^−1^ kg^−1^ (female) or ≥55 mL min^−1^ kg^−1^ (male), or a 5 km time trial of ≤20:00 min (female) or ≤18:30 min (male). For detailed inclusion criteria, please refer to clinicaltrials.gov/study/NCT05067426.

### Method details

#### Study design

The study was conducted over four weeks for all participants, starting with an 8- to 9-day pre-intervention phase, followed by a 7-day intervention period, and concluding with a 14-day post-intervention phase. The HSM group completed ten HIIT sessions within seven days. A second intervention group, the HSM+LIT group performed the same HIIT sessions including an additional 30 min (min) of low-intensity interval training (LIT) following each HIIT session. The CG continued with their usual training regimen. For the complete protocol, please refer to our study protocol.^4^ All physiological exercise tests (PET) and HIIT interventions were performed with running.

The study design and training protocol are outlined in Figures 1A and 1B. During the pre-intervention period, participants were familiarized with the equipment and underwent venous blood sampling at time point T0 (=baseline) and T1 as well as during and after the 7-day intervention period in the morning under resting conditions (either prior to an exercise at T2 [day two of the 7-day intervention period], T3 [day seven of the 7-day intervention period], T4 [three days after the 7-day intervention period], T5 [seven days after the 7-day intervention period] and T6 [14 days after the 7-day intervention period] or at day 4 [resting day] of the intervention and at +1 [one day after the 7-day intervention period]). All participants were equipped with a Global Navigation Satellite System (GNSS) watch (Forerunner 935, GARMIN, Kansas City, MO, USA) and a heart rate (HR) chest strap (HRM Pro, GARMIN, Kansas City, MO, USA) to monitor training duration and HR throughout the study period. Regular training during the baseline period was maintained, and participants underwent initial PET at time point T1 to establish individualized intensity ranges for the following HIIT sessions.^4^ In the post-intervention period, PET was repeated three times to assess changes in performance (at T4, T5, and T6). During the intervention period, four out of the ten HIIT sessions were monitored in the laboratory (two sessions at T2 and two at T3, respectively). The CG engaged in regular training and reported in the laboratory at T1, T4, T5, and T6 for PET. Throughout the study, all participants provided feedback through a questionnaire related to sleep, muscle pain, muscle soreness, well-being, and fatigue/exhaustion on a daily basis.

#### Physiological exercise testing

Prior to PET, participants were instructed to refrain from strenuous exercise, alcohol, and caffeine for at least 24 h. Study participants were asked to arrive in a fasted condition. Endurance performance was tested with a two-phase test on a treadmill (Saturn, HP Cosmos, Traunstein, Germany) with a breath-by-breath gas analyzer system (Quark CPET, Cosmed, Rome, Italy). Briefly, participants performed an incremental submaximal running test (with increases of 1.5 km/h every 3 min with 30-s rest for lactate sampling between each stage), followed by an 8 min recovery period, and a ramp test until voluntary exhaustion. The speed of the ramp test was based on the results of the incremental test (equal to the speed of the stage before the La increase of ≥1 mmol/L) and remains constant with a steady increase in slope (1.5% per minute), starting at 0% until voluntary exhaustion.^81^ VO_2max_ was determined during the ramp test as the highest 10 breath rolling average.

Heart rate was measured via HR chest strap Lactate was measured from capillary blood taken from the earlobe (Biosen S-line Clinic, EKF diagnostic GmbH, Magdeburg, Germany) throughout the incremental test to determine the lactate threshold. For the complete protocol, please refer to our study protocol.^4^

#### Intervention

All HIIT sessions started with a standardized 10-min low-intensity warm-up. Each HIIT session consisted of 5 interval bouts lasting 4 min at 90–95% of their individual maximum HR (HR_max_) interspersed with 2.5-min recovery periods at low intensity. This resulted in a total session duration of 40 min for HSM. Participants in the HSM+LIT group underwent the same HIIT sessions with an additional 30 min of LIT following each HIIT session, resulting in an additional 300 min of LIT volume across the intervention. The intensity of the LIT was conducted at a velocity associated with 1.5 mmol L^−1^ lactate, as determined at T1.

HIIT sessions were conducted in the morning (6–10 a.m.) and afternoon/evening (3–7 p.m.), with a minimum break of 5 h on days involving two HIIT sessions (Figures 1A and 1B). The intensity was continuously monitored through HR. Exercise intensity was prescribed based on individual HR zones, which were determined during T1. Treadmill speed was initially set to match the corresponding target HR zone. If necessary, speed was gradually increased during the session to reach the desired HR range. However, adjustments were made only within a reasonable range to ensure that participants could complete the session as planned. No reductions in intensity were implemented. Further details are presented elsewhere.^82^

Throughout the intervention, participants in both intervention groups were instructed not to undertake any additional training sessions beyond the prescribed training sessions. As this reflects realistic conditions and allows for adequate macronutrient fueling, participants were not required to arrive in a fasted state.

#### Blood parameters

Differential blood count (LYM, GR, WBC, MO%, LYM%, PLT, RBC, HGB, GR%, HCT, RDWCV, PDW%, MPV, MCH, MCHC, MCV) was determined from fresh whole blood samples using a Celltac MEK 6400 system (Nihon Kohden, Tokyo, Japan).

Serum cytokines (IL-2, IL-4, IL-5, IL-6, IL-9, IL-10, IL-13, IL-17A, IL-17F, IL-22, TNF-α, INF-γ) were analyzed simultaneously in duplicates with a bead-based immunoassay (Human Th Cytokine Panel [12-plex], Cat# 741027 and 741028, BioLegend, San Diego, California). Data acquisition was done by flow cytometry (Cytoflex, Beckman Coulter, California, U.S). The assay was performed with 5 μL of beads and 50 μL of undiluted serum per well.

CK and urea were determined with a light emitting diode photometer (Eurolyser CCA180, Eurolyser Diagnostica GmbH, Salzburg, Austria).

To quantify human ferritin and human transferrin an in-house sandwich ELISA was used. 96-well plates (F96 Maxisorp NUNC-Immuno Plate, Thermo Fisher Scientific, Roskilde, Denmark) were coated over night at 4°C with 2 μg/mL of a murine monoclonal antibody against human ferritin (Clone F23 [7A4], mouse IgG3, Cat# 4420-3010, Bio-Rad) or against human transferrin (Clone 507506, mouse IgG1, Cat# MAB5746, R&D Systems) in 1x phosphate buffered saline, 50 μL per well. Plates were blocked with 200 μL Tris-buffered saline, pH 7.4, 0.05% Tween and 0.5% bovine serum albumin for 2 h at room temperature (RT). Serum was diluted 1:50,000 (transferrin ELISA) and either used undiluted or diluted 1:2 (ferritin ELISA) and incubated for 2 h at RT, followed by 1 h incubation with a polyclonal rabbit anti-human ferritin (rabbit IgG, Cat# 12482-T16, Sino Biological) or anti-human transferrin (rabbit IgG, Cat# SIN-11019-RP02, Biozol) antibody diluted 1:5,000 or 1:1,000, respectively. Plates were then incubated with a horseradish peroxidase-conjugated recombinant monoclonal alpaca anti-rabbit IgG antibody (Clone IB21, Cat# CABT-L4215, Creative Diagnostics) diluted 1:10,000 for 1 h at RT. For the quantification, the plates were incubated in the dark with 50 μL 3,3′,5,5′-tetramethylbenzidine (KPL SureBlue TMB Microwell Peroxidase Substrate, Cat# 5120-0077, Lot#10560195, SeraCare 33 Life Sciences Inc.) for about 4 min. The reaction was stopped with 50 μL 1 N hydrochloric acid per well. The optical density was measured at 450 nm with a Tecan plate reader (Tecan Infinite M200 Microplate Reader, Männedorf, Switzerland). After each step, plates were washed three times with 100 μL 1x Tris-buffered saline, pH 7.4 and 0.05% Tween. All measurements were performed in duplicates, and average values of duplicates were used for further analysis. For data analysis 1,000 ng/mL of a purified native human ferritin (Cat# 4420–4804, Bio-Rad) or a recombinant human transferrin (Cat# ab83560, abcam) protein was titrated 1:4 until a concentration of 0.244 ng/mL was reached. The absolute serum concentrations were calculated via interpolation of a four-parameter logistic curve fit model in Graphpad Prism 9 (Version 9.1.2, Boston, USA).

#### Training load calculation

Training load was calculated based on HR data to establish a possible dose-response relationship between training load and biomarker concentrations. A three-zone training intensity model was determined based on a specific lactate concentration (1.5 mmol/L)^83^ obtained from PET at T1, separating zone 1 from zone 2, and a maximum HR percentage (90% HR_max_)^84^ obtained from the ramp test at T1, separating zone 2 from zone 3. Corresponding values for HR were determined with simple linear regression and the time spent in each zone was calculated for all training sessions. Following this, the Training Impulse (TRIMP) was computed by multiplying the time spent in each zone by its respective factor (zone 1 = 1, zone 2 = 2, zone 3 = 3), and subsequently summing these values up.^85^^,^^86^ To account for the influence of previous exercise sessions the TRIMP was weighted exponentially. For calculation of this exponentially weighted TRIMP (expTRIMP) the following formula was applied: load_today_ ∗λ + ([1 – λ] + EWMA_yesterday_) with λ = 2/(*N* + 1), determining the rate of decay and N being a time decay constant (here experimentally defined as three, five or seven days, i.e., 3-/5-/7-day exp TRIMP). The primary recorded training load was used to start with the calculation since no exponentially weighted moving average (EWMA) had been calculated for the day before (EWMA_yesterday_). This obtained value was then used as the EWMA_yesterday_ to continue the calculations with the above-mentioned formula.^87^^,^^88^^,^^89^

#### Self-reports

Throughout the study period, participants reported their subjective perception of sleep, muscle pain, muscle soreness, well-being and fatigue/exhaustion on a daily basis. Sleep was rated on a scale from 1 to 7, with 1 indicating very good sleep and 7 indicating very poor sleep.^90^ Muscle soreness, muscle pain and well-being were reported using a Visual Analog Scale ranging from 0 to 100 with 100 representing maximal soreness, pain and well-being).^91^ Additionally, fatigue and exhaustion were assessed on a scale from 1 to 10,^92^ with 10 representing the highest level of fatigue.

### Quantification and statistical analysis

Statistical analysis was performed using GraphPad Prism (Version 9.1.2, Boston, USA), R Studio (Version 4.4.1) and SPSS 27 (SPSS Inc., Chicago, IL, USA).

Mixed-effects model: Significant effects of intervention groups and time points as well as their interaction on logarithmic biomarker concentrations compared to CG were identified by restricted maximum likelihood mixed-effects analysis (fixed effects: group and time point summarized in pre-intervention (T0, T1), intervention (T2, 4, T3, +1) and post-intervention (T4, T5, T6); random effect: participants) (Figure 1D; Tables S1 and S2). Post-hoc analysis was performed as implemented in R Studio using emmeans for main effects (Time, Group) and interactions (Group | Time).

#### Area-under-the-curve (AUC) calculation

The AUC was calculated for all blood parameters during the periods from T0 to T1 (pre-intervention) and T5 to T6 (post-intervention). This calculation was used to assess chronic changes in biomarkers resulting from the intervention. Since similar trends in the response patterns pre- (AUC T0-T1) to post-intervention (AUC T5-T6) were observed in both intervention groups, HSM and HSM+LIT, we have decided to additionally pool the two groups.

#### Pearson correlation matrix

To investigate the relationship between biomarkers and training load (3-, 5- and 7-day expTRIMP) as well as with the self-reported assessment data, a Pearson correlation matrix and a network analysis were created with the absolute biomarker values and each self-reported assessment data from all participants of the study reported at rest prior to an exercise (T1, T2, 4, T3, +1, T4, T5, and T6), while for the expTRIMP the score for the previous day was used. Furthermore, a ranking from highest to lowest of the biomarkers' Pearson r of the 7-day expTRIMP and the muscle soreness score was created for all groups combined as well as for each group separately. Significant correlations were identified using a *p*-value threshold of 0.05, and only these were included in the final correlation matrix. Low and moderately significant correlations were defined as r < 0.29 and r > 0.3 and <0.49, respectively.^93^

To assess the connection between VO_2max_ and biomarkers, another Pearson correlation analysis was performed where VO_2max_ data of T1 were used for T0, T1, T2 and 4; VO_2max_ data of T4 was used for T3, +1 and T4 and VO_2max_ data of T5 and T6 were used only for the respective time point T5 or T6. Data of all time points of all 30 participants and all analyzed parameters were aggregated in the correlation analysis.

#### Network analysis

Network plots were generated to visualize the relationships between different variables using the *igraph* package. Edge weights in the network were determined based on the correlation coefficients, transformed for better visualization. The Fruchterman-Reingold layout algorithm was employed to arrange the nodes.

#### Threshold definition

To distinguish between measurement noise and meaningful changes, the standard deviation of each biomarker measured at T0 and T1 of all 30 participants was calculated to set a threshold (i.e., SD threshold). From a biological perspective, high inter-individual variability due to factors such as training status, nutrition, genotype or phenotype, and measurement errors frequently make reliable interpretation of obtained performance data difficult. Therefore, defining a threshold such as the smallest worthwhile change or confident intervals for meaningful post-intervention changes can be applied to aid in this situation.^94^ In the present study, the SD threshold was introduced as a concept to distinguish between measurement noise and meaningful changes.

#### Combined biomarker

In an exploratory approach, we aimed to identify a combined biomarker, which reflects the training load and the muscle soreness more sensitively than a single one. The blood biomarker with the highest positive (CK) and the highest negative (RBC) Pearson r calculated in the correlation matrix with the 7-day expTRIMP were used to calculate the ratio (logCK-to-logRBC) and were correlated with the 7-day expTRIMP determined the day before as well as with the visual analog scale for muscle soreness.
