# Supplementary material for: How intense is high-intensity interval training? Biomarker responses and associations with training load and fitness
Source: iScience. 2025 Oct 8;28(11):113738. doi: 10.1016/j.isci.2025.113738 (PMC12595005; doi:10.1016/j.isci.2025.113738)
Supplement: Document S1. Figures S1–S7 and Tables S1–S3 [file mmc1.pdf]

iScience, Volume *28*

Supplemental information

How intense is high-intensity interval training? Biomarker responses and associations with training load and fitness

Nils Haller, Hannah L. Widauer, Tilmann Strepp, Natalia Nunes, Julia C. Blumkaitis, Mario Wenger, Thomas Stöggl, and Lorenz Aglas

Supplementary Information

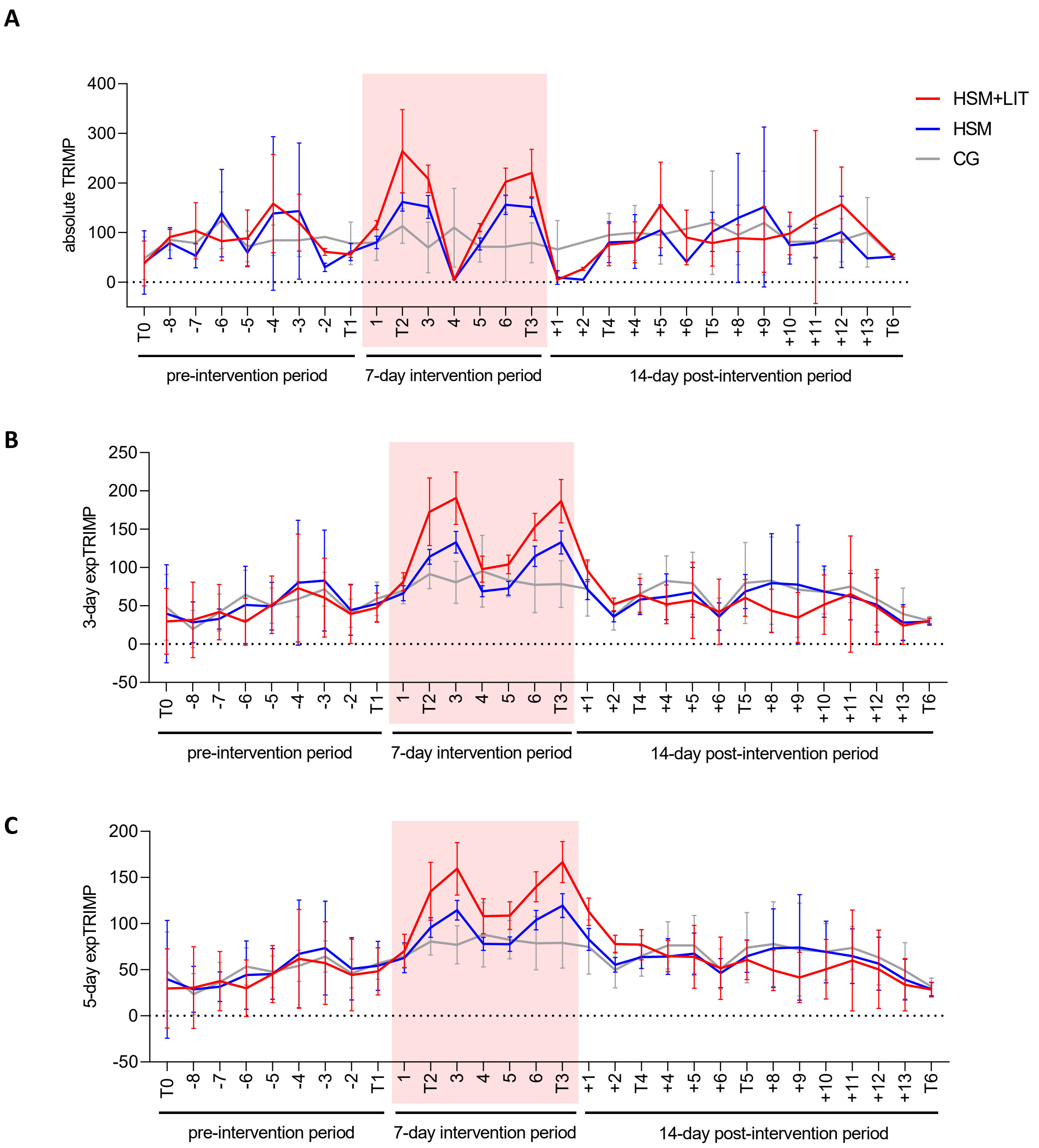

Figure S1: Training load monitoring during the study, related to Figure 1. (A) Absolute TRIMP values (B) 3-day expTRIMP and (C) 5-day expTRIMP. The TRIMP was weighted exponentially over the last 3 or 5 days to account for the influence of previous exercise according to the EWMA formula: $load_{today} * \lambda + ([1 - \lambda] + EWMA_{yesterday})$ with $\lambda = 2 / (N + 1)$ being a constant determining the rate of decay and N being a time decay constant (here set as 3 and 5 days). TRIMP, training impulse; expTRIMP, exponentially weighted training impulse; EWMA, exponentially weighted moving average; HSM, high-intensity interval training shock micro cycle; HSM+LIT, HSM and additional 30 min low-intensity training; CG, control group.

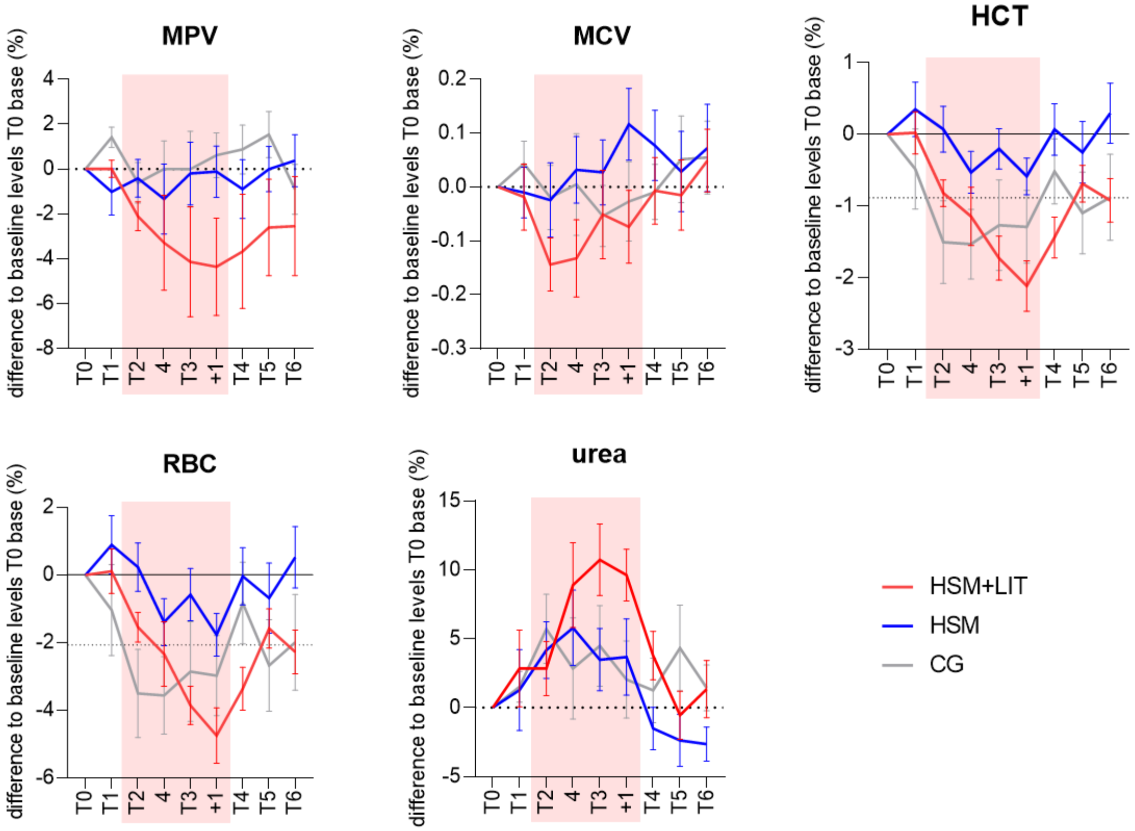

Figure S2: Percentage changes from baseline T0 for significant biomarkers (group*time). Data are shown as mean with SEM. Percentage differences compared to baseline T0 were calculated with the formula 100*(value-baseline)/baseline. Red area, blood collection time points during (T2, 4, T3) and one day after (+1) the 7-day intervention period. HSM (blue), high-intensity interval training shock micro cycle; HSM+LIT (red), HSM and additional 30-minute low-intensity training; CG (gray), control group; SD, standard deviation.

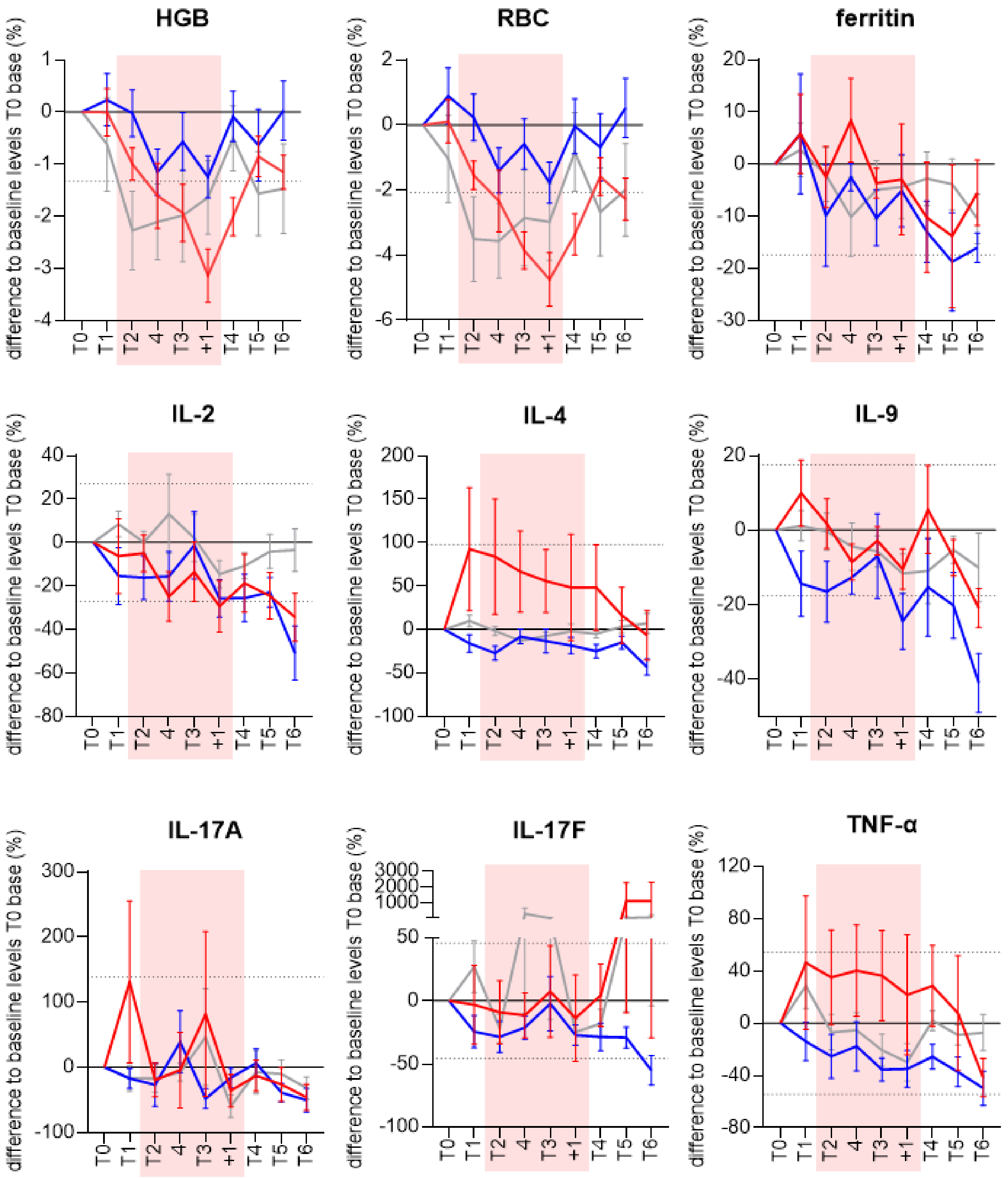

Figure S3: Percentage changes from baseline T0 for significant chronic effects (comparing T0-T1 and T5-T6) observed for the biomarkers. Data are shown as mean with SEM. Percentage differences compared to baseline T0 were calculated with the formula 100*(value-baseline)/baseline. For cytokines, absolute values were used in all calculations, while for the other parameters the logarithmic values were used. Red area, blood collection time points during (T2, 4, T3) and one day after (+1) the 7-day intervention period. Dotted lines represent the SD threshold calculated for biomarker values at T0 and T1 of all 30 participants. HGB: SD = 1.33, RBC: SD = 2.07, ferritin: SD = 17.38, IL-2: SD = 27.13, IL-4: SD = 97.93, IL-9: SD = 17.60, IL-17A: SD = 138.61, IL-17F: SD = 45.77. HSM, high-intensity interval training shock micro cycle; HSM+LIT, HSM and additional 30-minute low-intensity training; CG, control group; SD, standard deviation.

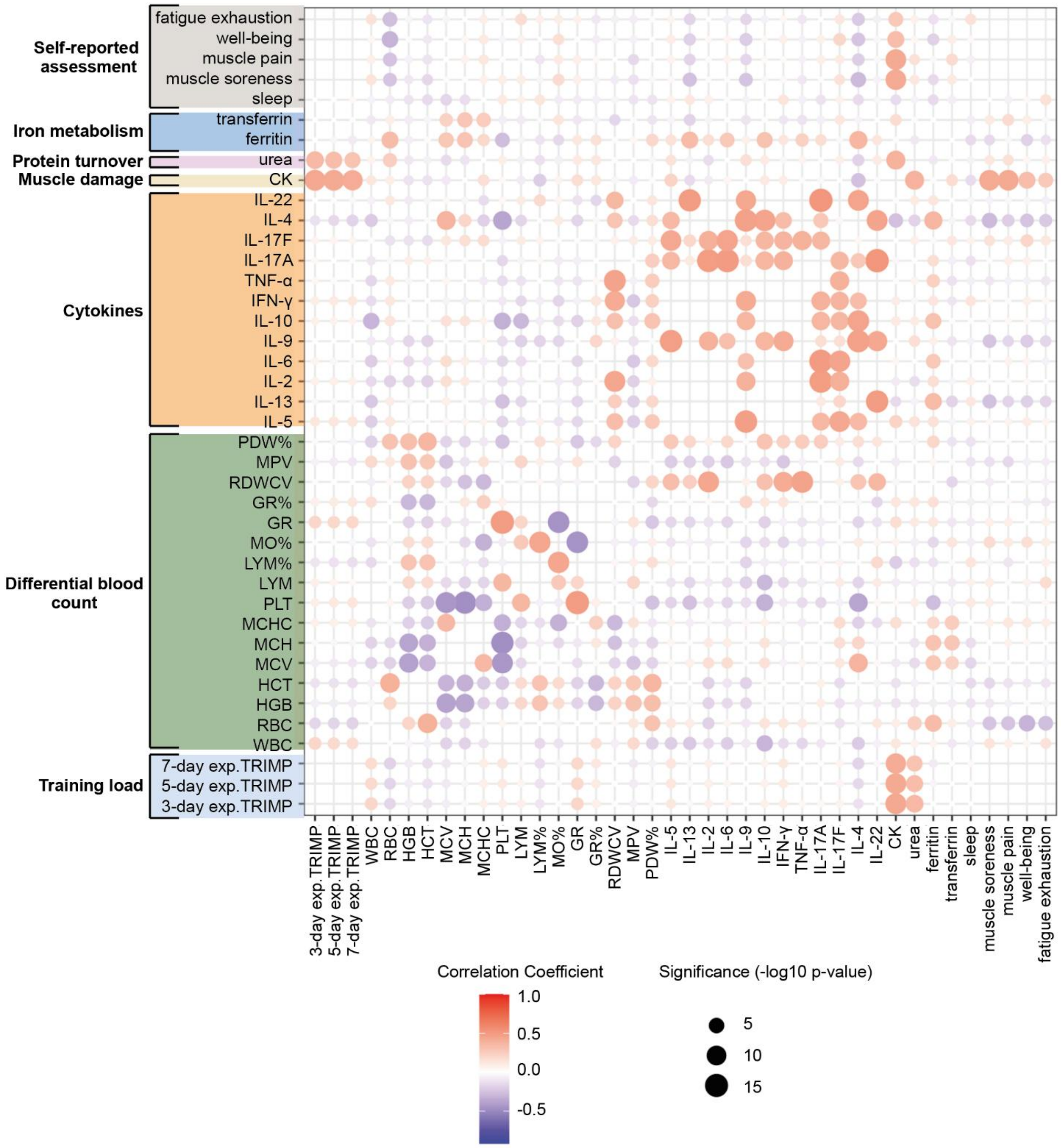

Figure S4: Evaluation of a combined biomarker that correlates with trainings load and muscle soreness related to Figure 4. Correlation matrix of all 32 blood biomarkers, training load (3-, 5- and 7-day expTRIMP) and the self-reported assessment dataset. Absolute data of all biomarkers and all participants were used for calculation of the matrix. Only correlations with $p < 0.05$ are displayed.

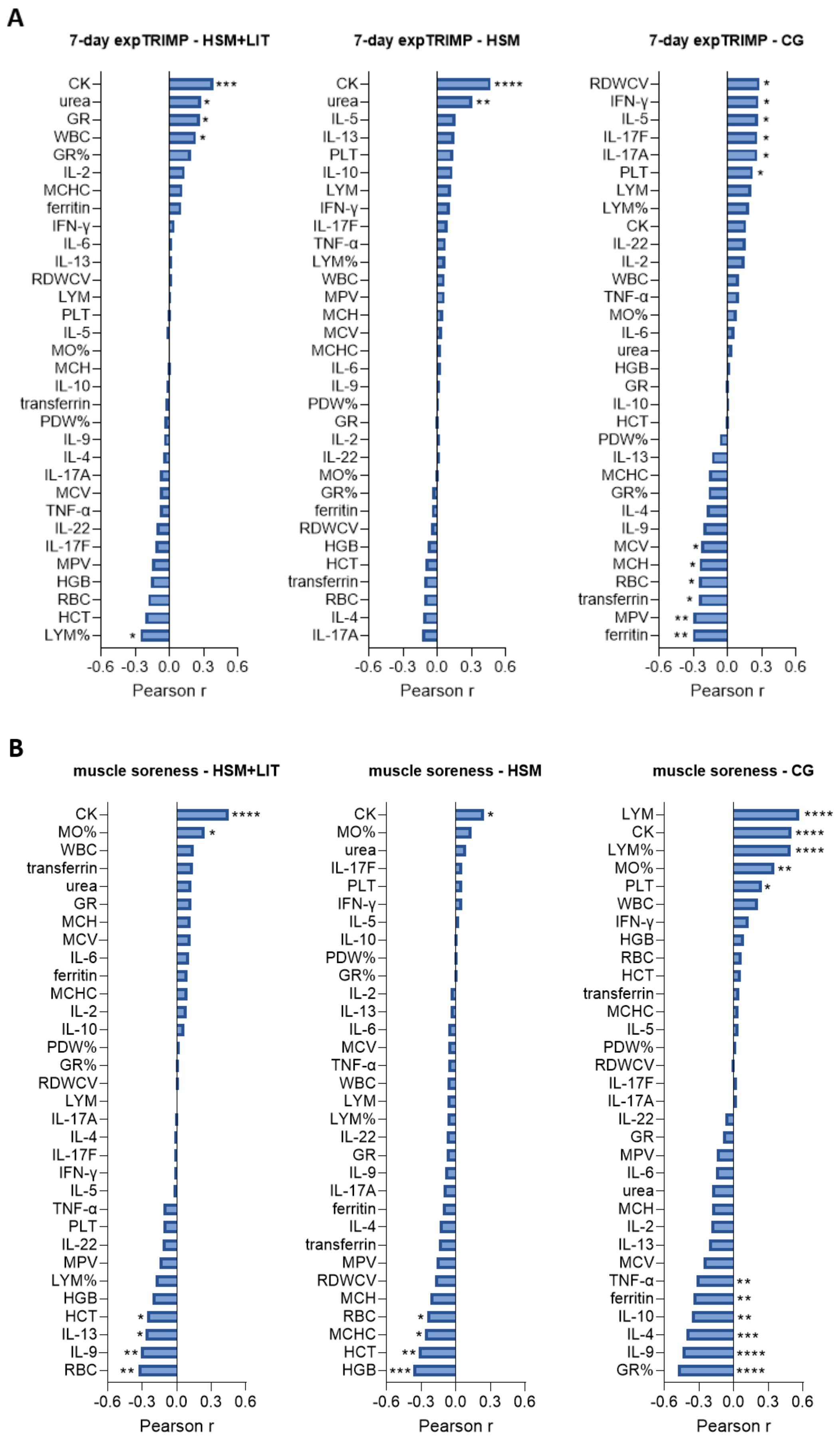

Figure S5: Ranking of correlations of the 32 biomarkers with the 7-day expTRIMP (A) and with the muscle soreness score (B), related to Figure 5. Absolute values of biomarkers were correlated with the 7-day expTRIMP calculated for the previous day or with the muscle soreness score on the same day. Statistical significance is given by $p < 0.05$ (*), $p < 0.01$ (**), $p < 0.001$ (***), $p < 0.0001$ (****). HSM, high-intensity interval training shock micro cycle; HSM+LIT, HSM and additional 30-minute low-intensity training; CG, control group.

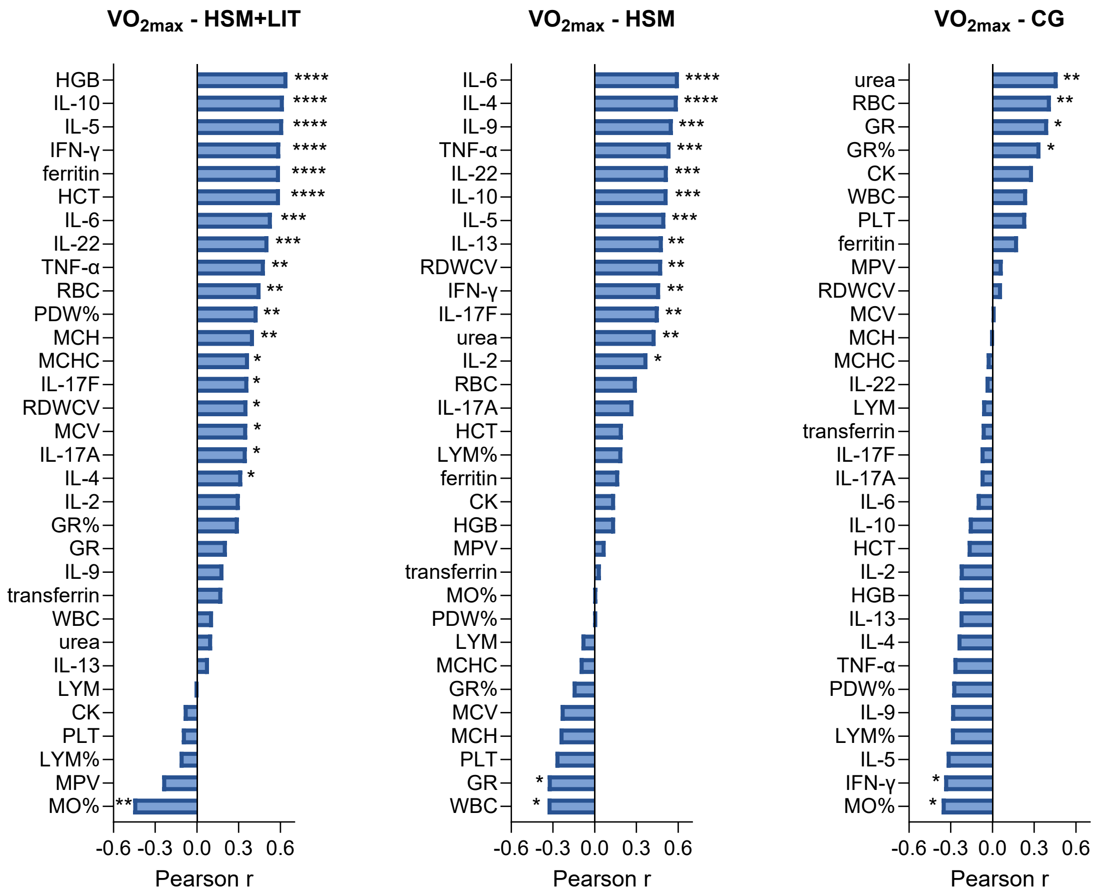

Figure S6: Ranking of correlations of the 32 biomarkers with VO_{2max}. Statistical significance is given by $p < 0.05$ (*), $p < 0.01$ (**), $p < 0.001$ (***), $p < 0.0001$ (****). HSM, high-intensity interval training shock micro cycle; HSM+LIT, HSM and additional 30-minute low-intensity training; CG, control group.

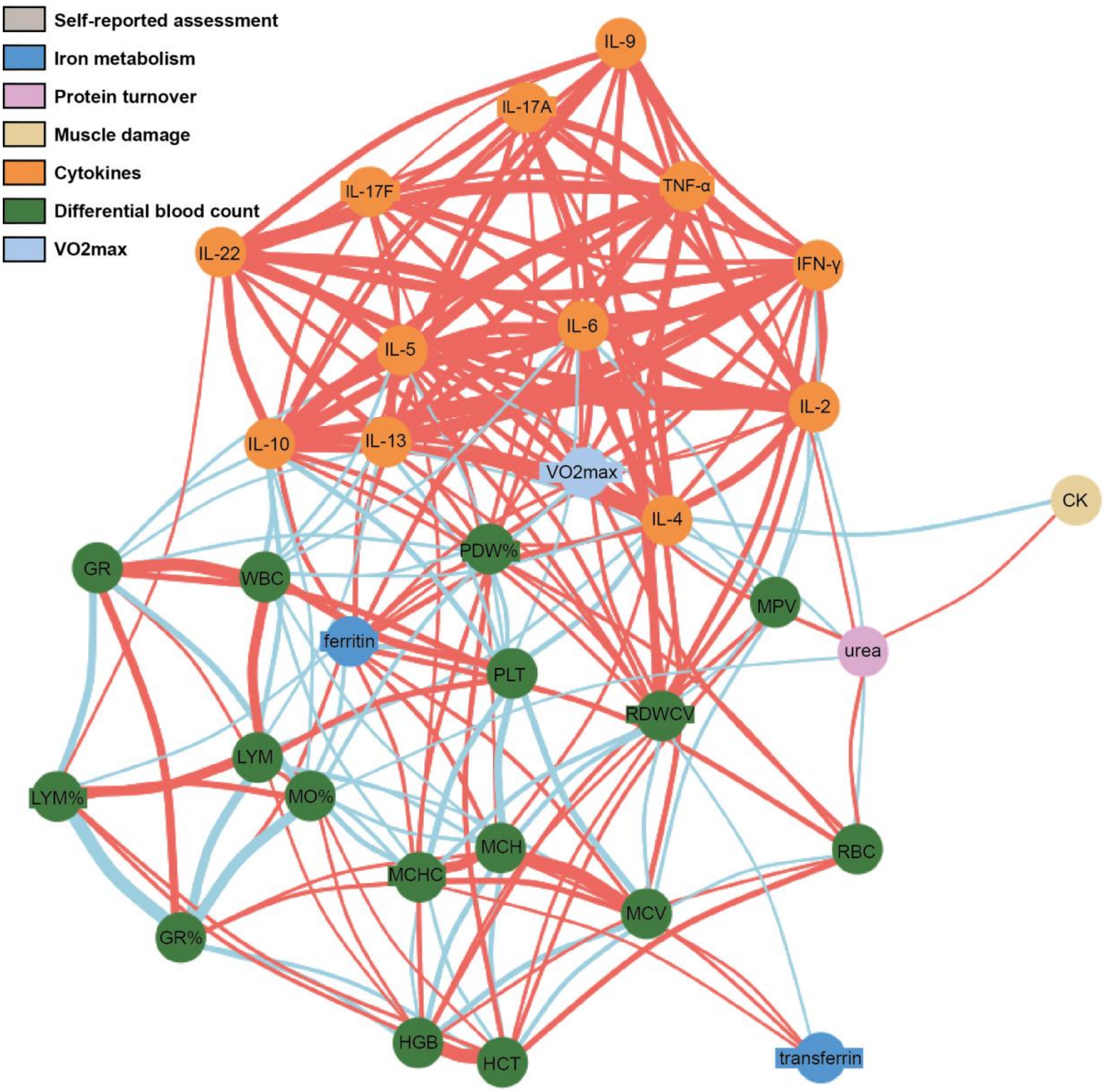

Figure S7: Interconnectedness of biomarkers and Vo$_{2max}$. Network including data of all 30 participants and all 32 biomarkers. Red lines display positive correlations, blue lines display negative correlation. Knots are only connected if significant correlations were observed.

Supplementary Tables

Table S1. Significant group*time interactions of all measured blood parameters and the 7-day expTRIMP compared to CG, related to the restricted maximum likelihood mixed-effects analysis stated in the STAR methods section. Fixed effects: group and time point, random effects: participants. Time points were summarized into pre-intervention (T0, T1), intervention (T2, 4, T3, +1) and post-intervention (T4, T5, T6).

Biomarker	Group	Time	Estimate	Pr(>|t|)	p value summary
RBC	HSM	Intervention	0.011	0.03645953	*
HCT	HSM	Intervention	0.012	0.016392	*
MCV	HSM	Intervention	0.002	0.02597286	*
CK	HSM	Intervention	0.139	0.03619976	*
RDWCV	HSM	Intervention	0.015	0.0000524	****
RDWCV	HSM	Post-Intervention	0.017	0.0000075	****
CK	HSM+LIT	Intervention	0.171	0.00970097	**
Urea	HSM+LIT	Intervention	0.071	0.0153712	*
MPV	HSM+LIT	Intervention	-0.023	0.02377554	*
RDWCV	HSM+LIT	Intervention	0.011	0.00195218	**
MPV	HSM+LIT	Post-Intervention	-0.023	0.02774358	*
LYM	HSM+LIT	Post-Intervention	-0.053	0.03765729	*
RDWCV	HSM+LIT	Post-Intervention	0.011	0.00338201	**
7-day expTRIMP	HSM+LIT	Intervention	0.354	0.000972749	***

Table S2. Significant time effects of all measured blood parameters and the 7-day expTRIMP compared to pre, based on the restricted maximum likelihood mixed-effects analysis stated in the STAR methods section. Fixed effects: group and time point, random effects: participants. Time points were summarized into pre-intervention (T0, T1), intervention (T2, 4, T3, +1) and post-intervention (T4, T5, T6).

Biomarker	Time	Estimate	Pr(>|t|)	p value summary
RBC	Intervention	-0.02	4.92E-08	****
HGB	Intervention	-0.021	1.15E-07	****
HCT	Intervention	-0.021	2.47E-08	****
Ferritin	Intervention	-0.067	0.02778789	*
IL-10	Intervention	-0.085	0.01713158	*
LYM	Intervention	0.041	0.01566387	*
RDWCV	Intervention	-0.006	0.01531065	*
RBC	Post-Intervention	-0.011	0.00604163	**
HGB	Post-Intervention	-0.011	0.00684217	**
HCT	Post-Intervention	-0.011	0.00290243	**
IL-9	Post-Intervention	-0.069	0.03034548	*
Ferritin	Post-Intervention	-0.068	0.03243791	*
IL-10	Post-Intervention	-0.081	0.03198701	*
RDWCV	Post-Intervention	-0.006	0.02238683	*
7-day expTRIMP	Intervention	0.19	0.011922086	*

Table S3. Overview of chronically altered biomarkers. The AUCs of the %diff values from pre-HIIT (T0-T1) and post-HIIT (T5-T6) intervention of each participant were calculated, and the median was compared within each group. The change from pre- to post-HIIT is given in percentage (%) and only significant changes are shown. Data is shown as median with SD. Upward arrows indicate increase, downward arrows indicate decrease. %diff, percentage difference from the baseline (T0); HSM, high-intensity interval training shock micro cycle; HSM+LIT, HSM and additional 30-minute low-intensity training; CG, control group; AUC, area under the curve.

Biomarker	HSM+LIT	HSM	CG	HSM+LIT & HSM
IL-2		↓ 33.4% ± 16.9		↓ 24.0% ± 38.0
IL-9		↓ 24.5% ± 20.1		↓ 11.2% ± 18.4
TNF-α		↓ 36.4% ± 29.8		↓ 28.1% ± 32.1
IL-17A		↓ 31.5% ± 37.0		
IL-17F		↓ 22.2% ± 25.2		
Ferritin		↓ 9.0% ± 7.2		↓ 9.2% ± 10.7
HCT	↓ 12.0% ± 7.9			↓ 7.6% ± 9.5
RBC	↓ 18.2% ± 8.5			↓ 9.8% ± 11.2
CK	↓ 11.0% ± 20.5			↓ 7.2% ± 17.1
HGB	↓ 11.5% ± 6.7		↓ 8.9% ± 17.5	↓ 8.5% ± 10.7
RDWCV			↓ 23.6% ± 13.7	↑ 15.4% ± 17.0
IL-4				↓ 20.7% ± 52.7
